# Community treatment orders: background and implications of the OCTET trial†

**DOI:** 10.1192/pb.bp.113.044628

**Published:** 2014-02

**Authors:** Tom Burns, Andrew Molodynski

**Affiliations:** 1 Department of Psychiatry, Warneford Hospital, Oxford

## Abstract

Community treatment orders (CTOs) were introduced into the UK despite unconvincing international evidence for their effectiveness. The Oxford Community Treatment Order Evaluation Trial (OCTET) is a multisite randomised controlled trial of 333 patients with psychosis conducted in the UK. It confirms an absence of any obvious benefit in reducing relapse despite significant curtailment of liberty. Community mental health teams need to seriously consider whether they should continue using CTOs or shift their clinical focus to strengthening the working alliance.

Community treatment orders (CTOs) have been introduced over the past 30–40 years in over 70 jurisdictions in an attempt to address the phenomenon of significantly disabled patients who present major challenges to supporting them in the community, so-called ‘revolving-door’ patients, in the era of deinstitutionalisation.^1,2^ Their early introduction was in the USA (where their use varies significantly between states) and then in Australia and New Zealand (where their use is highest). Several European countries have introduced CTO legislation, including Scotland in 2005 and England and Wales in 2008. The legislative structures have different names and despite differences in conditions, duration and eligibility, there are surprising similarities in how and for whom they are used.^1^ They generally target patients who have been compulsorily detained in the past, have limited insight into their illness and who are judged to be at high risk of relapse. The typical patient is most often male, diagnosed with schizophrenia, with a number of previous admissions, often under compulsion. The presentation is frequently one of self-neglect and isolation rather than risk to others. Community treatment orders rely on the provision for rapid recall to hospital for their enforcement. The most common requirements are adherence to medication and regular contact with the clinical team.

Community treatment orders were initially suggested in the UK by the Royal College of Psychiatrists in 1988 and then in a more detailed Council Report in 1993.^3^ That proposal was strikingly similar to the eventual legislation but required evidence of relapse on discharge from a previous compulsory admission and set an absolute limit on renewal of 3 years. Strong opposition was experienced both within and outside the College based on fears of force being applied in patients' homes (although this has been explicitly prohibited in all legislation) and skepticism about the justification for coercion with patients who are otherwise able to survive outside hospital. More recently, opposition has included concerns about the absence of any convincing evidence of their effectiveness.^1^

An earlier attempt to completely redraft the Mental Health Act 1983 based on capacity^4^ had been rejected by the government but an update was considered necessary. This was predominantly to deal with the ‘loophole’ of the treatability condition for personality disorder and to introduce a form of CTO. The amendment was introduced in 2007 and CTOs became available in England and Wales in 2008.

## The current state of the evidence

Churchill *et al* summarised the 72 studies available in 2006^1^ and a recent update by Maughan *et al* has identified another 18 studies.^5^ The evidence falls into three broad groups. Many early studies are descriptions of practice or uncontrolled outcome studies from which no firm conclusions can be drawn about effectiveness. The second group comprises case-control studies. These draw on existing administrative databases of patients on CTOs and use them as their own controls in before-and-after studies or match them with similar patients to compare their rates of admission to hospital following the imposition of a CTO. These studies yield conflicting results^5^ and are unable to confidently account for differences in availability of treatments. For example, the greatest reductions in hospital use were in New York but access to high-intensity case management was initially restricted to CTO patients.^6^ Although these studies do not give consistent results for rates of readmission, they generally find that CTO admissions are shorter.

The third group of studies contains randomised controlled trials (RCTs). These are clearly needed because of the limitations of case-control studies and their conflicting results. However, there have only been two, both in the USA and both over a decade ago.^6,7^ Neither found a reduction in the rate of admission.

## OCTET: rationale and design

The Oxford Community Treatment Order Evaluation Trial (OCTET) is a clinical effectiveness study of whether CTOs reduce the rate of relapse and readmission in patients with psychosis. It was designed as a rigorous, multisite, single outcome, pragmatic RCT. It aimed to isolate the effect of the CTO from other potential differences in management and to test its impact when added to naturalistic, high-quality community mental healthcare.^8^ To reduce excess variability and for practical reasons, OCTET was restricted to adults of working age (18–65 years) with a psychotic diagnosis and without forensic restrictions. We asked community mental health teams from collaborating services to submit all patients that they were proposing to put on CTO on discharge from Section 3 of the Mental Health Act (i.e. not to cherry-pick) and to offer similar levels of clinical contact irrespective of the arm of randomisation. There were no other constraints on clinical practice. The primary outcome (as in the two previous RCTs) was the proportion readmitted over the next 12 months. Secondary outcomes included time to readmission and length of hospital stay. The protocol was registered with *The Lancet* and the detailed analytical plan fixed before any data were examined.

We conducted the trial immediately after the introduction of the legislation, as international experience indicates that clinicians quickly form strong opinions about CTOs and clinical equipoise is soon lost. We asked teams to aim for weekly contact if at all possible. This derived from three key considerations. First, it is the level of contact that assertive outreach teams generally provide for their most vulnerable patients. Second, the principle of reciprocity suggests that depriving patients of their liberty should be matched by a significant compensatory clinical response.^9^ Last, three contacts per month was the median level in the North Carolina study and one which they considered essential to good outcomes.^6^

Our original aim was to randomise between CTO and discharge to voluntary care but current legislation made this impossible. The trial encountered very specific ethical considerations and required a detailed legal opinion before it was permitted.^10^ This introduced two important constraints. First, at the point of randomisation the alternative to a CTO had to be Section 17 leave (Mental Health Act) – patients could not be simply discharged to voluntary care. Section 17 leave allows patients currently detained in hospital to be allowed out for periods of hours or days without any change in their legal status. It is a purely clinical decision and does not involve legal considerations. In the past, Section 17 leave has been misused by being prolonged over considerable periods but the new legislation specifically proscribes such practice. To ensure that Section 17 leave was not used in such a way as an ersatz CTO, we only recruited from teams who undertook to use it as indicated in the Mental Health Act *Code of Practice*^11^ and not to engage in extended Section 17 leave. Second, we could not insist that patients remained in the arm of randomisation for the whole 12 months – clinicians had to remain free to change their minds about management.

## OCTET results

We approached all National Health Service mental health trusts within a day's journey of Oxford. Of these, 46 agreed to cooperate and arranged Research and Development approval and 32 were eventually recruited to the study. Recruitment varied significantly between trusts (from 1 patient to 32) and between teams. We over-recruited our target sample (333 patients) and obtained 100% of primary and secondary outcome data. Our randomisation was successful – there were no baseline differences in the two groups. Our patients also closely matched the descriptions of CTO patients in other studies.^1^ The level of clinical contact was the same in both arms (a median of two and mean of three contacts per month). Section 17 leave was generally short term (median of 8 days) compared with the CTO median of 183 days. Thus any differences we found could therefore be confidently attributed to the effect of the CTO.

Our findings are stark and uncomplicated. There was absolutely no difference in the proportion readmitted (36% in both arms) nor in the time to readmission (a hazard ratio of 1.00). Total length of hospital stay was not significantly different at 41.5 days for CTO patients and 48 days for Section 17 leave patients. So our study confirms the previous two RCTs – CTOs do not achieve their principle stated purpose of reducing relapse and readmission.

## Limitations

No RCT is perfect and ours has some important limitations. The most striking was that over 20% of patients did not get the treatment to which they were randomised. Overall, 13 patients were never discharged from hospital (7 on a CTO, 6 on Section 17 leave), 35 CTO patients never had the CTO imposed and 40 Section 17 leave patients went directly to CTO. Many of these violations reflect changes in clinical condition during the period required for considering and imposing a CTO. However, those going direct from Section 17 leave to CTO are less explainable. In a clinical effectiveness trial like OCTET, a number of protocol violations are to be expected but this is certainly more than one would want. We conducted a sensitivity analysis excluding these violations and there are still no significant differences in outcomes apart from length of hospital stay, which was shorter in CTO patients (*P* = 0.035). The primary outcome of proportion readmitted was 35% CTO *v*. 39% Section 17 leave.

The second important limitation is that we were unable to estimate how many patients could have been eligible for the trial. There remains a possibility that the study is drawn from a narrow range of potential patients, although the clinical features of those recruited give no reason to believe this. The study sample had high rates of previous detained admissions, self-harm and criminal convictions, suggesting considerable morbidity. However, it did not include elderly patients who might be considered to be more likely to be influenced by a legal constraint or forensic patients who might be more used to responding to sanctions.

## Conclusions and implications

None of the three RCTs testing CTOs has found a significant benefit in reducing relapse and readmission. Both the North Carolina study and OCTET are sufficiently powered to conclude that CTOs do not have any effect over a year's follow-up. All three RCTs have some limitations, but these vary between them. Their fundamental finding does not. The weight of evidence must surely be that CTOs, despite considerable infringement of patients' liberty, are not effective in well-established and functioning mental health services.

It is possible that there may be some specific clinical subgroups for whom CTOs do work, but there is nothing in the current evidence to support this or to indicate who they are. Establishing that this is the case will require trials at least as rigorous as OCTET. The proposition that complex community interventions may take more than 12 months to have their effect has generally not been confirmed in other studies.^12^ It seems very unlikely that this would be the case with absolutely no effect in the first year.

The conduct of our trial provided some disquieting observations that call for attention. The first was the striking lack of continuity of care experienced by these patients. Less than a quarter had only one consultant for the whole year – most had more and some up to five as they passed between community mental health team consultants, in-patient consultants and even crisis team consultants. For a patient group selected for needing consistent and intensive monitoring this is surely unacceptable. We were also dismayed by the number of consultants who told us they could no longer enter patients into our study because they ‘had seen with their own eyes’ that CTOs work. It is simply not possible to ‘see with your own eyes’ a probabilistic outcome which takes months to manifest itself.

Community treatment orders were introduced in the UK by a government which knew that there was no convincing evidence of their effectiveness. There were other considerations in the balance – restoring public confidence in mental health services, reducing unacceptable variations in practice and ensuring adequate monitoring of community compulsion. However, OCTET has changed this balance. There is strong evidence that liberty is being substantially curtailed without any obvious clinical benefit to justify it. Indeed, OCTET underestimates the extent of this curtailment of liberty as it is censored at 12-month follow-up – many patients on CTOs beyond 6 months (i.e. 50% of them) will go on to repeat renewals.

If we believe that psychiatry should be an evidence-based profession and clinical trials are a worthwhile exercise, then we should not ignore the findings. More and better trials are undoubtedly indicated and we have suggested limitations in OCTET that deserve attention. However, these are unlikely to occur in the very near future. Our findings are strong and should be taken seriously. We believe that there should be a moratorium on the further imposition of CTOs (and consideration of those already in place) other than in research settings unless and until convincing evidence of their effectiveness is obtained. It may be time to cease pursuing risk-based coercive interventions (which lack evidence) and refocus our efforts into restoring enduring and trusting relationships with patients.
